# Typology and characterization of the agricultural productive units in the NE Amazonian region of Ecuador

**DOI:** 10.5455/javar.2024.k762

**Published:** 2024-03-31

**Authors:** Armando Vinicio Paredes Peralta, Santiago Alexander Guamán Rivera, María Gabriela Tobar-Ruiz, Marcelo Eduardo Sánchez-Salazar, Pablo Danilo Carrera Oscullo, Leonardo Fabio Medina Ñuste

**Affiliations:** 1 Universidad Estatal Amazònica, Tena, Ecuador; 2 Escuela Superior Politécnica de Chimborazo (ESPOCH) Sede Orellana, El Coca, Ecuador; 3 Scientific Researcher, Riobamba, Ecuador

**Keywords:** Amazonian region, productive units, productive system, smallholders

## Abstract

**Objective:**

Many studies have observed different characteristics among productive systems in the rural territories of Latin America. Therefore, understanding and characterizing them while they function plays an essential role in determining their relationship between development and environment. A study has been conducted in the Orellana province of NE Ecuador to determine their typology and then classify them according to the variables that describe their main traits or attributes using cluster analysis (CA).

**Materials and Methods:**

A survey was structured to investigate physical, productive, environmental, as well as socioeconomic character variables, which were subsequently applied to a random sample of the 5,963 agricultural productive units (APUs) through face-to-face contact with producers during an *in situ* visit to their farms.

**Result:**

The CA allowed us to identify three typologies of APUs in the Orellana Province. The first has been Type 1, which is denominated as the most conventional (40%), while Type 2 uses more efficient natural resources but represents an amount of only 9.4%. In contrast, type 3 (50.6%) depends on a significant part of local or national development programs.

**Conclusion:**

All groups indicated some peculiarities in common, as there were marked differences in the use and distribution of land as well as production methods among them. Consequently, this pioneering study allowed us to identify different production methods. Therefore, we encourage local and national governments to establish policies for natural resource conservation in such high-diversity zones.

## Introduction

The last decade has been seen as one in which the rural territories of Latin America have experimented with great changes in their production systems [1,2]. These changes are most likely related to practices of inadequate use and exploitation of natural resources. Furthermore, there is a substantial amount of scientific evidence that the major impact nowadays could be climate change [3–5]. Therefore, this global event may also affect the biological processes of productive systems due to their complex and sensitive functionality.

Producers in tropical landscapes regularly experiment with very complex interactions between forest dynamics, production forms, levels of productivity, and marked integrations of the people, including ethnicity and socioeconomic issues [1,6]. Nonetheless, Thornton et al. [5] observed that many tropical or subtropical regions, due to their characteristic edaphoclimatic, temperature, and heterogeneity of households, can experiment with very large. Thus, it needs a certain outlook for a correct equilibrium among all components within a productive system due to the high level of sensitiveness of the biotic elements, as a measure to achieve the sustainability of these producers’ livelihoods.

Ecuador, situated on the northwestern edge of South America, has about 256,370 km^2^ of surface and is geographically divided into four areas: the Andean highlands, the coastal lowlands, the Amazon basin, and the Galapagos archipelago. It is a country of strong biodiversity and geodiversity and plenty of cultural diversity, which allows for the provision of numerous ecosystem services [7–9].

The Amazonian Region (RA) covers 116,588 km^2^ which represents some 45.47% of the country´s surface [10,11]. The average rainfall varies between 2,000 and 5,000 mm annually, with an average temperature of 24°C. This region is formed of six provinces: Zamora Chinchipe (9.1% of the RA), Morona Santiago (20.6%), Pastaza (25.4%), Napo (10.7%), Orellana (18.6%), and Sucumbíos (15.5%).

The Ecuadorian Amazon consists of a variety of ecological reserves, such as Cayambe-Coca, Faunistic Production of the Cuyabeno, Biological Limoncocha, and Cofan-Bermejo. Furthermore, there are two important national parks: the Sumaco-Napo Galeras and the Yasuni. The latter reserve corresponds to the biggest protected area of Ecuador, hosting thousands of animals and a rich flora as a global biodiverse hot spot. It also contains a very important freshwater reserve and has been recognized to have a global climate regulatory function as a greenhouse gas sink [7,12–14]. In this zone, five sectors of biogeography and twenty-five ecosystems have been identified [15]. In addition, in this zone, high tree densities have been determined, which include bushes, flooded and inundated forests, as well as semideciduous forests. Furthermore, diverse economic, political, and social activities have been developed lately. These include colonization, migration, oil and timber extraction, as well as large oil palm plantations [16,17]. This has led to numerous, well-documented social and environmental impacts due to extensive agriculture, cattle ranching, and vast oil extractions [7]. Furthermore, as this region is especially vulnerable to biodiversity losses due to peaks of species diversity, 19 different ecosystems, a third of its protected zones coincide spatially with oil block concessions [16,18]. Consequently, it is a very fragile ecosystem, whose consequences could be severe and adverse, with irreversible impacts on its capacity to provide ecological services in the future.

Hence, a fundamental point to consider when developing a planning process for decision-making processes are biological and territorial factors, since the outcomes and consequences of such actions may be irreparable [11,19,20]. Currently, several researchers have affirmed that typology studies are very adequate tools to enhance the understanding of decision-making behaviors as well as the strategic progress of individual producers and holdings [20,21–23]. Twongyirwe et al. [23] as well as Signorelli [24] have indicated that this is one approach to designing targeted interventions that adequately address the needs of different types of farmers. Therefore, the typology tries to demonstrate the maximum heterogeneity between the types while maximizing homogeneity within individual types or categories [25,26]. In addition, the selection of differentiation criteria depends on the objective and typology where data are available [21].

The province of Orellana forms part of the RA, representing 18.6% of a total of 45.47% and having a total population estimate of 157,520 inhabitants [27]. This zone has demonstrated a complex social composition, experimenting with severe changes since the 1960s as a result of several laws of agrarian reform, subsequent colonization, and the beginning of upcoming hydrocarbon production [16,28]. Few studies determined that agriculture expansion is conducted mainly by small-scale farming systems but lacked to demonstrate the not fully understood impact of smallholders. Hence, the absence of information about the productive systems has not permitted us to understand the deeper structure of this involvement. Consequently, these large limitations during the design and execution of the public policies developed by national or international institutions may be linked to a lack of knowledge of the functioning of rural territories and their productive systems.

Based on the aforementioned, the current study has been performed to determine the productive typology and then classify it according to different variables that will allow it to describe its main traits or attributes. Therefore, we initiated a survey that has been elaborated very well and structured with variables of all kinds—social, economic, and productive—as well as validated by a group of experts in each area. The primary aim has been to characterize, describe, and interpret the productive systems to decipher their functioning and establish the different relations. Typologies are usually elaborated to understand the farming systems [26,29], observed land use and level of intensification [30,31], technology adoption [32], livelihood strategy [9,33], vulnerability to climate change [4,5,21], and environmental assessment [34,35]. The outcome of the study may serve as a resource for a defined database for any type of governmental intervention in this province, while helping to enhance the livelihoods of the local producers and allow more farm systems to become sustainable.

## Materials and Methods

This studied site attracts great attention and interest for forthcoming research due to a variety of reasons, which include forest conservation, ancestral settlements inhabited by indigenous populations, as well as some half a century of colonization, which represents a severe and very potential threat to the given biodiversity [33,36].

### Study area

*Location:* The current study has been performed in the province of Orellana, located in the northern Ecuadorian Amazon basin, which is distributed in four districts (cantons), named Francisco de Orellana, Loreto, Joya de los Sachas, and Aguarico. According to the Gobierno Autónomo Descentralizado Provincial de Orellana (GADPO) [37] and González Marcillo et al. [38], this province has 21.730 km^2^ of surface area (18.6% of the total 45.47% of the RA). The climate in the region is characterized by humid tropical rainforest [39]. The average rainfall is about 2,942 mm annually, with a temperature of 29.7°C during the year. Instituto Nacional de Estadisticas y Censos- Encuesta de Superficie y Producción Agropecuaria Continua (INEC ESPAC) [27] has estimated a population of some 157.520 for the year 2018. The ethnic groups are comprised of Native Amerindians (Kichwa, Shuar, and Waodani) at 31%; mestizos (the mixed descendants of Spanish colonists and indigenous Amerindians at 57.5%); and the small Afro-Ecuadorian minority at 4.9% [40].

### Livelihoods

Agricultural land use in the Orellana province, according to estimates from INEC-ESPAC [27], has indicated a total surface area of 606,307 ha, which is arranged in mountains and forests (485.039 ha; 80%), permanent crops (43.582 ha; 7.2%), other uses (28.049 ha; 4.6%), cultivated pastures (25.162 ha; 4.2%), natural pastures (19.034 ha; 3.1%), as well as transitory crops and fallow (4959 ha; 0.82%).

The population density is relatively low, with some eight inhabitants per square kilometer [20]. There are strong differences between indigenous practices and colonos; while the firsts have a concept mostly comprised of subsistence farming systems, the colonos are more diverse and include activities such as subsistence farming and small-scale intensive farming systems [41].

In this sense, Lòpez et al. [7] and GADPO [37] indicated that the main activities for the generation of income producers in the Amazon region are concentrated in agriculture (56.5%) and livestock (10%), while 30% are under a mixed production system (agriculture-livestock). However, all these activities employ intensive systems in the natural resources and workforce with very low productivity and rentability levels. On the other hand, the forest or agroforest activities that take advantage of the forest resources only represent about 1.4% of all Amazonian producers.

### Farm survey data

The working set of data considered in the current study was the number of agricultural productive units (APUs) registered by the GADPO [37,39] and INEC-ESPAC [27]. In the province of Orellana, there are 5,963 APUs that comprise a total of 250,172 hectares. In conclusion, the APUs have a farm size range of 20 to 50 hectares, maintaining perennials and transitory crops, pastures, as well as forest reserves.

While elaborating the questionnaire, first, a multidisciplinary team was formed with producers and local scientists with a variety of expertise (soil science, agronomy, rural sociology, veterinarians, economists, and statistics in agriculture) with the objective of designing a format to investigate variables representing physical, productive, environmental, and socioeconomic traits of the APUs, based on previous studies [42–44]. The second step of the survey was pre-tested on producers to ascertain how they employ certain production practices to validate in situ and check the suitability of the questions according to the methodology described by Lòpez-Roldàn and Fachelli [45].

### Variable selection

Through the survey applied to the selected population, we chose variables that had more relevance in the explanation of the functioning and structure of the productive systems in the province of Orellana. Consequently, we selected 31 variables that should be able to explain these factors. These shared several characteristics, like a low coefficient of variation such as <30%, as listed in Table 1, which means low communality and, therefore, adequate homogeneity of data. Hence, the retained variables were (a) socioeconomic, which includes age, education level, farm and livestock incomes as well as off/non-farm income; (b) physical elements, which include farm total surface, mountains and forests; (c) pastures, which include natural and cultivated; (d) permanent crops; (e) transitory crops; and (d) fallow; and finally (c) the productive elements, which include a split of paddocks, grazing rotation, animal composition, backyard animals, working animals, cocoa and coffee crops, age of calf at weaning, fattening beef cattle time, weight total fattening phase, and milk production [26].

### Methods of typology construction

The principal component analysis (PCA) was applied to reduce the dataset, which allowed for the visualization and processing of a high-dimensional dataset [46]. Therefore, this technique, which linearly transforms many independent variables into smaller ones, is probably the most adequate to be used [47].

The criteria to decide how many principals’ components (PCs) to keep were based on the Kaiser’s-Guttman Rule. Thus, PCs exceeding an eigenvalue of 1.00 were retained [21]. Likewise, the variables with low or intermediate factor loads were excluded according to the recommendations of Kuwahara et al. [22] and Bidogeza et al. [31]. Consequently, in the present study, loadings superior to or equal to 0.50 have been considered for interpretation in Table 2.

### Cluster analysis (CA)

Retained factors from PCA were used in CA using Ward´s hierarchical procedure. This method is frequently used by many researchers as it minimizes the variance within the cluster. The dendrogram and multidisciplinary expert team of the zone were employed to select an optimal number of clusters using discriminant analysis of variance [31]. Finally, the data was analyzed through SAS version v. 9.4. (SAS Institute Inc., Cary, NC). Differences were declared at *p* < 0.05 and tendencies at *p* < 0.10.

Furthermore, CA is often used to determine classifications within complex databases as well as to ascertain and establish relationships between groups. Another fundamental point is to explain the different typologies that allow us to detect differences in variance between clusters. Finally, this method comprises several steps, such as the review and selection of variables, factor analysis, and CA, as well as its interpretation.

## Results

### Categorization of APUs

When analyzing our data with the PCA procedure, we obtained five PCs, which explain around 63.14% of the variability in the dataset (Table 2). Furthermore, the first obtained PCs explain about 20.59% of the variability in the data. Hence, the first component (PC1) was apparently related to the explanation of variables such as the total size of a farm, the split of paddocks (size), and other animals used for working in the farms (e.g., horses and mules). Contrastly, the PC1 was less related to variables of permanent crops and the total weight of animals fed on paddocks.

The second component (PC2) obtained narrow relations with farm surface, cultivated pastures, herds’ total size, and total period destined to fatten *Bovidae.* Likewise, the third component (CP3) was linked to subsistence farming, raising minor species such as poultry and pigs for their own feeding and some others, which they commercialized in local markets. The fourth and fifth components, PC4 and PC5, respectively, had variables such as the age of the producers and transitory crops.

### Identified typologies

**Figure 1.**
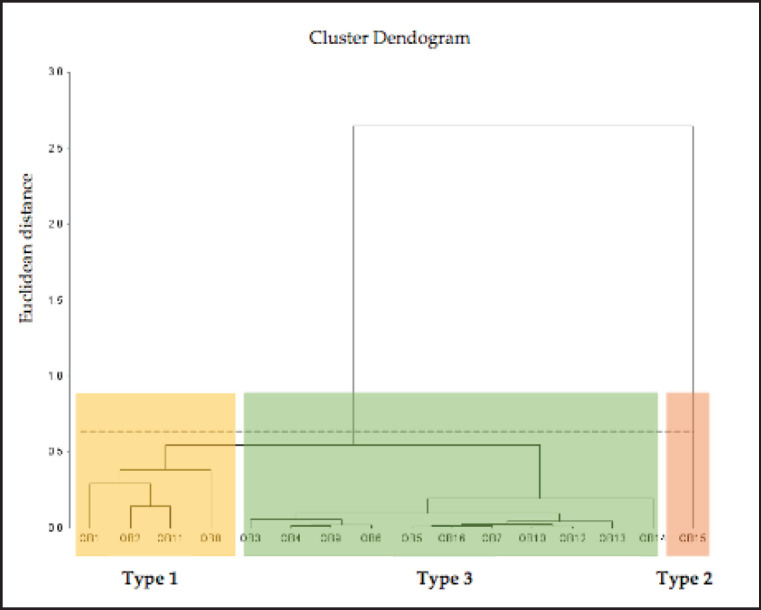
Dendrogram of three identified typologies. The cluster is based on agglomerative hierarchical clustering with Euclidean distance as the similarity measure and Ward’s linkage strategy.

Using Ward´s hierarchical procedure, we identified three clusters based on Euclidean distances. Therefore, Figure 1. shows a dendrogram with the different typologies.

### Type 1

The type 1 typology includes moderately large farm surfaces, being one very particular and conventional group, while it represents around 40% of the sampled APUs (*n =* 76). This type is the most conventional found in this province, where their farms have some 31 ha on average and whose age of producers is approximately 49 years old. Their referent to the educational level is 76.2% having basic education (first six years), 14.3% receiving secondary education, and 9.52% having not received any education. Besides, they have distributed their APUs with forests (41%), pastures (46%), and permanent crops (10%), while a low amount (3%) are only transitory crops.

**Table 1.** Description of variables used for typology constructions.

Variable	Unit	Mean	SEM	Min.	Max.	CV
***Socioeconomic*** Age Education level Income Crop’s sales Livestock sales (TLU)^1^ include all reported. Off/non-farm income^5^	year year USD/month USD/month USD/month	48 6 15 8 30	1.7 0.4 1.5 0.5 3.6	27 0 5 3 15	71 12 22 11 42	3.5 6.7 10 6.3 12
***Physical*** Farm total surface Mountains and forests Pastures, include (natural and cultivated) Permanent crops Transitory crops and fallow	ha ha ha ha ha	30.5 11.0 10.1 2.82 6.17	2.4 1.9 1.3 0.3 1	5 0 1 0 0	68 50 48 10 39	7.9 17.3 12.9 10.6 16.2
***Productive*** Split of paddocks (size) Grazing rotation Animal composition *(Bovidae)* Backyard animals (poultry and pig) Working animals *(Equus caballus* and *Equus africanus asinus*) Cocoa Coffee Age of calf at weaning Fattening beef cattle time (steers and heifer calves) Weight total fattening stage Milk production	ha day TLU_1_ TLU TLU ha ha month month kg/ L/d	1.77 33.0 7 28 2 1.9 0.8 4.5 8 72.7 2.5	0.3 3.8 0.9 5.0 0.4 0.2 0.1 0.4 1.8 17.3 0.4	0 0 0 0 0 0 0 0 0 0 0	10 160 25 212 9 8 3 10 36 350 10	16.9 11.5 12.9 17.9 20 10.5 12.5 8.9 23 24 16

Source: Author’s analysis of the 2017 to 2019 survey data; ^1^TLU = Tropical livestock Unit corresponds to an animal ruminant of estimated weight 250 kg; SEM: standard error of the mean. CV = coefficient of variation

This type is characterized by having large paddocks of >2 ha with long grazing periods (rotation of 54 days between grazing, on average), while the herd size average is about 10 bovines (tropical livestock unit (TLU)). As the surface area occupied by pastures is around 46% in relation to the total hectares of their farms, we identified forage species C_4_ such as guinea grass or Saboya (*Panicum maximum)*, *Brachiaria brizantha* cv. Marandu, and *Brachiaria humidicola*. These forage species are usually cultivated as monocultures without association with any leguminous species. Although in their paddocks there are dispersed trees of low density (<38 tree/ha−^1^, on average), the environmental services for the protection of their animals against heat, stress, or high precipitation are incalculable. This typology uses working animals such as horses and mules (1 TLU, on average); likewise, they raise a low number of poultry and pigs as backyard animals. Also, it should be emphasized in this type that due to deficient management and inadequate use of land for achieving the total weight of its animals and subsequent commercialization, they need about 31 months, reporting a live weight of some 264 kg. As they have mainly mestizos’ breeds *(Bos indicus)* adapted to tropical climates, milk production lacks importance in this group, with some 5 l/d. We might highlight in this typology APUs that sow and cultivate permanent crops such as coffee (*Coffea arabica L.*) and cocoa (*Theobroma cocoa*) whose surfaces are not more than 2 ha on average. By contrast, they all year sow, harvest, and sell transitory crops, such as maize (*Zea mays*), rice (*Oryza sativa*)*,* tuber crops such as cassava (*Manihot esculenta*)*,* and plantains (*Musa AAB*). Consequently, the economic income in this typology, according to their reply and case studies, could possibly be 200 USD per month. Therefore, these types of APUs are extensive productive systems with deficient management of their natural resources. We can clearly see the expansion of the agricultural frontier with marked reductions of forests, basically subsistence farms.

**Table 2.** Eigenvalues and percentage variance are explained by the five principal components (PCs) using PCA.

PC	Eigenvalue	Variance (%)	Cumulate variance (%)
1	3.29	20.59	20.59
2	2.30	14.41	35.00
3	1.84	11.50	46.51
4	1.39	8.70	55.20
5	1.27	7.94	63.14

**Table 3.** Least square mean of variables in the different typologies of agricultural productive Units in Orellana province, Amazonian Region of Ecuador.

Variable	Agricultural productive Units			SEM	Difference
	Type 1	Type 2	Type 3		*p*-value
Age of the producers (year)	49.0	46.4	47.3	3.7	0.868
Farm total surface (ha)	30.8	38.4	28.9	4.9	0.531
Mountains and forests (ha)	12.7	21.6	19.6	4.2	0.330
Surface destinate to pastures (ha)	14.2^x^	7.0^z^	9.1^y^	2.6	0.099
Permanent crops (ha)	3.1	2.8	2.9	0.6	0.929
Transitory crops (ha)	7.1	7.6	5.6	2.1	0.720
Paddock size (ha)	2.3	1.8	1.4	0.4	0.165
Grazing rotation (days)	53.8^a^	27^b^	19.4^b^	2.4	0.001
Size herds (Bovidae) TLU	9.79	8.6	7.2	2.0	0.513
Mules and horses TLU	1	0	1	0.2	0.743
Backyard poultry TLU	22.4	44.4	35.2	10.1	0.343
Backyards pigs TLU	4	0	4	0.5	0.643
Age of calf at weaning (month)	6.4^a^	3.4^b^	6.5^a^	0.3	0.001
Period of fattening (month)	31^a^	24^b^	22^b^	1.2	0.004
Total weight at fattening (kg)	264	297	263	15.9	0.553
Milk production (kg/day)	4^b^	7^a^	5^b^	0.5	0.004

SEM, standard error of the mean; TLU, Tropical livestock Unit, corresponds to an animal ruminant of estimated weight 250 kg.

### Type 2

APUs have a high presence of forests, a minor surface area of permanent crops, and rational management of their resources, but this typology constitutes only 9.4% (*n =* 18).

In general, like the prior group (Type 1), the age of producers is 46 years old. Likewise, 20% of these producers have received basic education, while 80% have finished secondary education (12 years on average). The farm surface has 38 ha, on average, in which the presence of forest occupies this stratum (56%), transitory crops (18%), and then pastures (18%); instead, there are barely (7.2%) permanent crops.

Although this type is small compared with the first typology (40% *vs.* 9.4%), they use their natural resources more efficiently. Likewise, they harvest transitory crops such as maize *(Z. mays*), rice *(O. sativa),* tuber crops such as cassava (*M. esculenta*), and plantains *(Musa AAB).*, which occupy 18%, but these producers have notable commercial perception.

In this group, pastures are cultivated, such as guinea grass or Saboya (*Panicum maximum*), *Brachiaria cv. humidicola brizantha* Marandu, as well as shrub species, with the purpose of improving animal nutrition. We highlight species such as *Gliricidia sepium* and Gray (*Tithonia diversifolia*). Furthermore, their paddocks have (2 ha, on average), in which they are grazing nine bovine TLU, whose rotation grazing is every 27 days. In addition, on this small typology, we can see producers with phenotypes of animals as a result of crosses with mestizo bovines *(B. indicus)* with cattle breeds specialized for beef and milk *(Bos taurus).*

These actions have been conducted to improve the weight of their animals (24 months) with an average live weight of 297 kg. This typology diversifies its production between livestock, crops, and the sale of minor species as well as other extra farm activities (total incomes of 450 USD per month on average), but they are also very conscientious about the degradation and loss of natural resources. Therefore, this group of APUs, according to our results, constitutes particularly important for the conservation and sustainability of these fragile and sensible present ecosystems. Our team hopes to be able to show how these APUs help to develop a balance among all biotic components within the productive systems.

### Type 3

Little APUs, in relation to the farm’s total surface, constitute about 50.6% (*n =* 96). Representing small productive systems (29 ha, on average) of their farms with high-density forests, they have distributed the use of land as follows: forests (66.76%), natural and cultivated grasses (31%), permanent crops (11%), and transitory crops (19%). In fact, these properties have been obtained with crops already established as well as quite hectares occupied with pastures such as guinea grass or saboya *(Panicum maximum)* and *B. brizantha* cv. Marandu and *Brachiaria humidicola*. Referent to transitory and permanent crops, these APUs nearly have no differences from anterior APUs (Type 1 and Type 2), which have been reported to have a surface area of 3 and 6 ha, respectively.

The predominant crops are coffee (*C. arabica* L.), cocoa (*T. cocoa*)*,* as well as fruit trees of low density: guava fruit (*Psidium guajava* L.), and guama (Inga edulis). Also, they cultivate maize, rice, cassava tubers, and the root crop taro (*Colocasia esculenta* (L.) Schott). In the same way, they use pastures to feed half-breed bovines (*B. indicus*) herd size TLU 7, with grazing rotation every 19 days, obtaining a total weight of 263 kg in a period of 22 months. Normally, they sell a few minor species as poultry and pigs (total monthly income: 325 USD, on average). Consequently, this typology works hand in hand with statistical programs to develop productive systems that are more efficient and sustainable. However, the producers most of the time evaluate all programs for the economic profit that they generate. In many cases, the departure of a lot of participants would lead to the loss of economic resources for state organizations and severe damage to biodiversity as well as their production systems.

## Discussion

### Productive system patterns

The CA allowed us to identify three typologies of APUs in the Orellana Province. Using principal components analysis (PCA), the data shows the total size of the farm, the split of paddocks, the animals used to work in the farms (horses and mules), the time devoted to fattening steers and other Bovidae categories, subsistence farming, backyard species, livelihood socioeconomic status, and finally commercialization methods are among the largest sources of variation. Therefore, in Orellana Province, the APUs have been classified and characterized as follows: Type 1, denominated as the most conventional (40%), Type 2 apparently uses natural resources more efficiently but only represents 9.4%, whereas Type 3 (50.6%) depends on a great part of local national development programs. However, it is important to mention that all these groups were mainly differentiated by surface and land use, animal husbandry as ruminants, and backyard species.

According to Weltin et al. [25], this differentiation plays an important role in establishing a relationship between nature and society as a way to use their natural resources more adequately, defining and preserving their strategies for future generations over long time periods. In this sense, several works have been developed in Latin America to carry out the typification of productive systems; they have revealed important information that nowadays is useful when we should adopt or make decisions. Contrasting this evidence, Zhunusova et al. [1] stated that local economies could be conditioned by factors such as deforestation and conservation strategies. So, different traits among productive systems could be the result of extrinsic and intrinsic factors [21]. Supporting this, Rojas-Downing et al. [4] and Cuevas-Reyes and Rosales-Nieto [48] have cited that in many countries, the mixed systems are synergistic between crops and livestock. Consequently, identifying typologies offers a synthetic assessment tool of farm management indicators as an integrated set rather than as a single indicator, thus highlighting links between the different indicators. At the level of Ecuador, despite the wide diversity of productive systems, the majority of farmers develop their agriculture activities as subsistence activities with low theoretical levels, so the classification methods and typification allow them to perform studies with large data sets, and it’s used by many researchers to know the variables that are needed to help explain most events that occur within productive systems. By summarizing and recognizing the structure and practices usually employed, it will be possible to propose alternatives for improving productive systems and making them more sustainable.

### Land use

The RA, despite its higher biodiversity, has shown an intense change due to the accelerated rate of deforestation, migration, and exploitation of oil [49]. In Ecuador, Torres et al. [50] observed that small livestock farmers annually destinate big forest surfaces for conversion on pastures. Contrary to this result, in Orellana, despite type 1, there was more forest extension than in other typologies; however, this group showed an increase in deforestation rate and an advance of the agricultural frontier. Therefore, natural habitats have been converted to pasture as a final use of land. For this reason, the physical, chemical, and biological properties of soil have shown a process of degradation [51], with potential repercussions for water degradation, loss of nutrients, changes in patterns of biological cycles, and climatic changes [17]. Consequently, the most important effect on the use of land is the natural carbon cycle, increasing greenhouse gas emissions [52].

Other important results were that the forest has high relevance in all the groups described. However, apparently, type 2 is smaller than types 1 and 3 and takes the forest component more seriously in their farms. Typologies 1 and 3 are less efficient with respect to their distributed land, and they are very dependent on local and national entities to develop activities. Supporting this, Lessman et al. [18] showed that deforestation has been occasioned by agricultural colonization, encouraging new ways and patterns of population growth in the Amazon. Finally, we stated that all typologies are dedicated to agricultural activities as well as cattle ranching, but with clear differences between them in their use and land distribution. Therefore, from a sustainable viewpoint, we should lead towards these more productive systems with a high level of environmental conscience. Besides this, at the level of Ecuador, policies should lead to zones with productive potential, whereas they restrict those in ecologically sensitive areas, as recommended by Verburg et al. [53].

### Forestry and livestock

In this study, the distinct groups described do not follow a similar pattern of production. Each typology uses ways and techniques to produce and make the majority of their natural resources dependent on their socioeconomic status. This first study in Orellana Province showed that having high bovine numbers does not necessarily ensure greater productivity levels. In fact, the bovine type 1 showed a lower body weight gain than the other typologies studied. Besides this, it is important to emphasize that animal performances were conditioned by grazing management. We evidenced that type 1 used mature pastures due to high grazing frequency and high defoliation intensities, so this led to greater methane emissions due to the low nutritional forage quality [41]. There is enough scientific evidence to state that livestock contributes to climate change due mainly to land usage [17]. According to Borja et al. [10], Ecuador has lost 12.5% of its original Amazonian forests (12.120 of 96.073 km^2^) and is second after Brazil in the ranking of countries with higher deforestation rates. In the case of Ecuador, livestock husbandry is vital to both subsistence and economic development in the entire Amazon region. Although cattle raising is present in all the identified typologies, it plays a key role in the structure of productive systems. Nevertheless, deforestation due to livestock production constitutes the main problem in tropical regions. Therefore, we should apply actions that help both climate change adaptation and mitigation. To achieve it, it will have to be done through diversification of production patterns and the responsible exploitation of all resources.

Many authors have already mentioned the importance of studying and understanding the diversity of the farmers to propose more sustainable alternatives with the use of clean technologies that allow for the provision of a range of ecosystem services and to improve their quality of life [46]. A previous study determined that rural development policies are implemented without considering these aspects [17]. To support this, Sellers et al. [9] observed changes in demographic behavior, land use, forest cover, and living conditions in the North Amazon of Ecuador. This research has evidenced that a great number of producers are very dependent on local or national programs. So, they do not have a clear, suitable productive system that allows them greater productivity and to handle the sustainability of their natural resources. For this reason, this research is important since we can differentiate between three productive systems and understand how they interact with land use decisions. Based on this evidence, it opens up a huge field of study for trying to understand the different interactions between climate change and livestock.

## Conclusion

Our findings confirm the existence of three important typologies in the province of Orellana in the Ecuadorian Amazon. Although all groups have some features in common, there are also marked differences in land use and how they are distributed. Therefore, their production methods are different, as are their socioeconomic and environmental status. Consequently, the variety of identified farms, through our typology, may form a basis for prioritizing existing policies and targeting future interventions in a specific farming system. These results are fundamental at the local level since they are able to be adopted by the national government or other entities for proposing more adapted agricultural policies to their conditions to improve their quality of life and take care of the existing natural resources.

## List of Abbreviations

APUs, agricultural productive units; GADPO, Gobierno Autónomo Descentralizado Provincial de Orellana; GHG, greenhouse gas emissions; INEC ESPAC, Instituto Nacional de Estadisticas y Censos- Encuesta de Superficie y Producción Agropecuaria Continua; PCA, principal component analysis; RA, Amazonian region; SEM, standard error of the mean; TLU, Tropical livestock Unit.
